# Future career clarity and bedtime procrastination among college students: a self-regulation perspective

**DOI:** 10.3389/fpubh.2026.1842996

**Published:** 2026-08-17

**Authors:** Linxuan Huang, Mengyao Yan, Wendi Sun, Linsen Dai, Yuxuan Gou, Tongtong Li, Yunfei Duan, Lei Shi, Di Liu

**Affiliations:** 1 School of Automation, Harbin University of Science and Technology, Harbin, China; 2 School of Public Health, Peking University, Beijing, China; 3 School of Humanities and Management, Zhejiang Chinese Medical University, Hangzhou, Zhejiang, China; 4 School of Health Management, Guangzhou Medical University, Guangzhou, China; 5 Department of Sociology, School of Ethnology and Sociology, Minzu University of China, Beijing, China; 6 School of Marxism, Harbin Medical University, Harbin, China; 7 School of Computer Science and Technology, Harbin University of Science and Technology, Harbin, China; 8 Philosophy and Social Sciences Key Laboratory of Guangdong Higher Education Institutes for Health Governance Based on Big Data Utilization, Guangzhou Medical University, Guangzhou, China; 9 Guangdong Hong Kong Macao Greater Bay Area Medical and Health Industry High Quality Development Rule of Law Guarantee Research Center, Guangzhou, China

**Keywords:** bedtime procrastination, exercise, future career clarity, learning satisfaction, learning stress

## Abstract

**Background:**

Bedtime procrastination is common among university students and is associated with insufficient sleep and a range of adverse physical and psychological health outcomes. Previous studies have primarily focused on individual factors, such as self-control, mobile phone addiction, and negative emotions, while the interrelationships among future career clarity, learning experiences, exercise, and bedtime procrastination remain largely unexplored.

**Objective:**

Based on self-regulation theory, this study aimed to examine the association between future career clarity and bedtime procrastination among university students. It further examined whether learning satisfaction and learning stress statistically mediated this association in sequence, and whether exercise moderated the association between learning satisfaction and bedtime procrastination.

**Methods:**

A survey was conducted among 22,983 college students in Northeast China using an online questionnaire combined with respondent-driven sampling. Data were analyzed using partial correlation analysis, mediation effect testing, and moderation effect testing, with effect sizes and confidence intervals (CIs) estimated using the bootstrap method.

**Results:**

Partial correlation analysis revealed a significant negative correlation between future career clarity and bedtime procrastination (*r* = −0.282, *p* < 0.001). Mediation effect testing showed that the total indirect effect of future career clarity on bedtime procrastination was significant (effect = −0.1024, 95% CI [−0.1092, −0.0958]), with learning satisfaction and learning stress serving as mediators individually and jointly constituting a chain mediation pathway. Moderation effect analysis indicated that exercise significantly moderated the relationship between learning satisfaction and bedtime procrastination (*β* = −0.0135, *p* < 0.05). The mediation effects were significant for both the low exercise group (simple slopes = −0.1816, LLCI = −0.1966, ULCI = −0.1667) and the high exercise group (simple slopes = −0.2177, LLCI = −0.2467, ULCI = −0.1887), with the negative predictive effect being stronger in the high exercise group.

**Conclusion:**

Future career clarity not only negatively predicts bedtime procrastination among college students directly but also exerts an indirect influence through the serial mediating effects of learning satisfaction and learning stress. Exercise plays a positive moderating role in this pathway. Grounded in self-regulation theory, these findings elucidates the relationships among goal setting, emotional monitoring, and behavioral adjustment, providing both a theoretical foundation and practical guidance for universities to reduce college students’ bedtime procrastination through multidimensional approaches such as career planning education, stress management, and the promotion of physical exercise.

## 1 Introduction

Adequate sleep is of paramount importance for maintaining human health (1). In recent years, sleep medicine and issues related to sleep disorders have garnered significant attention from both the medical community and general public. This trend is partly attributable to a growing body of research indicating that sleep insufficiency and sleep disorders can negatively affect the function of multiple physiological systems. If left unaddressed, these issues may precipitate or exacerbate a variety of serious diseases (2). Against this backdrop, bedtime procrastination—an important behavioral factor influencing sleep onset and maintenance—has attracted widespread concern.

Bedtime procrastination refers to the failure to go to bed at the intended time in the absence of external interfering factors and is highly prevalent across different populations and age groups (3). This phenomenon is particularly prominent among college students. According to an international survey conducted by Peltzer et al. (4), approximately 10.4% of university students across 26 countries experience severe difficulties falling asleep at night. The factors influencing this phenomenon are relatively complex, encompassing multiple aspects such as poor economic conditions, off-campus residence, mental health problems, high-risk behaviors, inadequate social support, and poor academic performance (4).

In China, the continuous expansion of higher education has intensified the academic and competitive pressures faced by college students, making their sleep problems increasingly severe. Existing research indicates that bedtime procrastination is highly prevalent among Chinese college students, manifesting as a series of issues including delayed sleep onset, prolonged sleep latency, insufficient total sleep time, and reduced sleep efficiency. Furthermore, these sleep problems are significantly correlated with psychological disorders such as depression and anxiety (5). In addition, upper-year students face additional pressures from career planning and job competition, which further exacerbate their bedtime procrastination behaviors, posing greater challenges for intervention efforts (6).

Learning satisfaction refers to individuals’ subjective evaluations of their learning environment, learning processes, and learning outcomes, reflecting the extent to which their learning experiences align with their personal expectations and developmental goals (7). Higher learning satisfaction generally indicates that individuals derive a stronger sense of meaning, competence, and positive affect from learning activities, whereas lower satisfaction may be accompanied by emotional exhaustion and other adverse psychological experiences. Previous research has shown that emotional exhaustion among university students diminishes academic satisfaction while exacerbating academic procrastination. This suggests that negative learning experiences may not only reduce students’ satisfaction but also impair their capacity for behavioral self-regulation (8). In addition, Hendriksen et al. (9) found that poorer sleep quality was associated with less favorable mood, lower perceived academic performance quality, and lower role satisfaction, while stress and fatigue significantly predicted impaired academic functioning. Collectively, these findings indicate that learning experiences, emotional states, fatigue, and sleep are closely interconnected. However, previous studies have primarily examined learning satisfaction in relation to depression, emotional exhaustion, academic performance, and academic procrastination, while its association with bedtime procrastination as a specific health-related behavior remains insufficiently understood. This gap in literature warrants further investigation into the potential relationship between learning satisfaction and bedtime procrastination among university students.

Another factor that could be associated with bedtime procrastination among university students is learning stress. Learning stress refers to the psychological burden experienced by individuals when confronted with academic tasks, examination demands, performance expectations, and time constraints (10). When students perceive that their academic demands exceed their available coping capacity and resources, they may experience anxiety, fatigue, rumination, and emotional exhaustion. Longitudinal research has shown that, as academic terms progress and academic demands increase, university students exhibit significant reductions in sleep duration, sleep quality, and heart rate variability, together with increases in symptoms of anxiety and depression. These findings suggest that persistent stress within academic contexts may affect psychological health, sleep-related outcomes, and autonomic nervous system functioning (11).

Existing research has generally treated sleep quality or sleep duration as an outcome of learning stress, paying comparatively less attention to whether learning stress influences the behavioral process through which students voluntarily postpone bedtime. According to self-regulation theory, sustained efforts to cope with academic demands may deplete limited cognitive and emotional resources, making it more difficult for students to resist smartphone use, video viewing, or other immediately rewarding activities at the end of the day (6, 12). Learning stress may also create a need to compensate for insufficient leisure and autonomy during the daytime by extending one’s personal time at night. Although such behavior may provide temporary relief from stress, it can also make it more difficult to follow one’s intended bedtime schedules. Therefore, the present study proposes that higher levels of learning stress could be associated with greater bedtime procrastination among university students.

In addition to learning-related experiences, students’ perception of their future careers may also play a role in shaping bedtime procrastination. Future career clarity refers to the extent to which individuals possess a clear understanding of their future career goals and the actions required to achieve them. Clear career goals may help students recognize the connection between their current learning activities and future career development while strengthening goal orientation, action planning, and proactive developmental behaviors. Previous research has shown that career-goal clarity and proactive career behaviors predict positive student outcomes. When career goals are less clear, active career exploration and proactive behaviors may also provide a degree of psychological protection (13). Research informed by goal-setting theory and the demand–resources framework further suggests that clear and personally meaningful career goals encourage students to proactively engage in career preparation (14). In addition, individuals with more salient future work selves are more likely to engage in behaviors that are consistent with their anticipated occupational identities. Longitudinal evidence indicates that a salient future work self predicts subsequent proactive career behaviors. Although direct evidence regarding the relationship between future career clarity and bedtime procrastination remains limited, career clarity may constitute an important motivational and goal-related resource. Accordingly, the existing literature suggests that higher future career clarity may be associated with lower levels of bedtime procrastination among university students.

Another factor that may be relevant to bedtime procrastination is regular physical exercise. Exercise is an important lifestyle behavior that influences both physical and psychological health, as well as sleep-related behaviors. Rather than focusing exclusively on the physiological effects of exercise on sleep quality, a self-regulation perspective emphasizes its potential role in reducing stress, regulating emotions, and improving behavioral control. Research among Chinese university students has shown that physical activity is negatively linked to both anxiety and bedtime procrastination, whereas anxiety is positively associated with bedtime procrastination. Physical activity and anxiety have also been found to mediate the relationship between mobile phone addiction and bedtime procrastination (15). Another study among university students further reported that exercise mitigates bedtime procrastination and might indirectly reduce bedtime procrastination by enhancing self-control and decreasing mobile phone addiction (16). On this basis, the existing literature suggests that that higher levels of exercise may be associated with lower levels of bedtime procrastination among university students.

Overall, previous research on bedtime procrastination has predominantly focused on individual self-control, chronotypes, mobile phone addiction, anxiety, and fatigue, giving comparatively limited attention to university students’ learning environments, perceptions of future career development, and everyday health behaviors. Although previous studies have separately documented the associations between learning satisfaction, learning stress, future career clarity, exercise, and a range of psychological, academic, and sleep-related outcomes, few studies have incorporated these factors into a single analytical framework or examined their independent relationships with bedtime procrastination. In particular, direct empirical evidence linking learning satisfaction and future career clarity to bedtime procrastination remains scarce. To address these gaps, this study examines how learning satisfaction, learning stress, future career clarity, and exercise relate to bedtime procrastination among university students—approaching the issue from four complementary perspectives: learning experience, academic demands, future-oriented goals, and health behavior.

**Figure 1:**
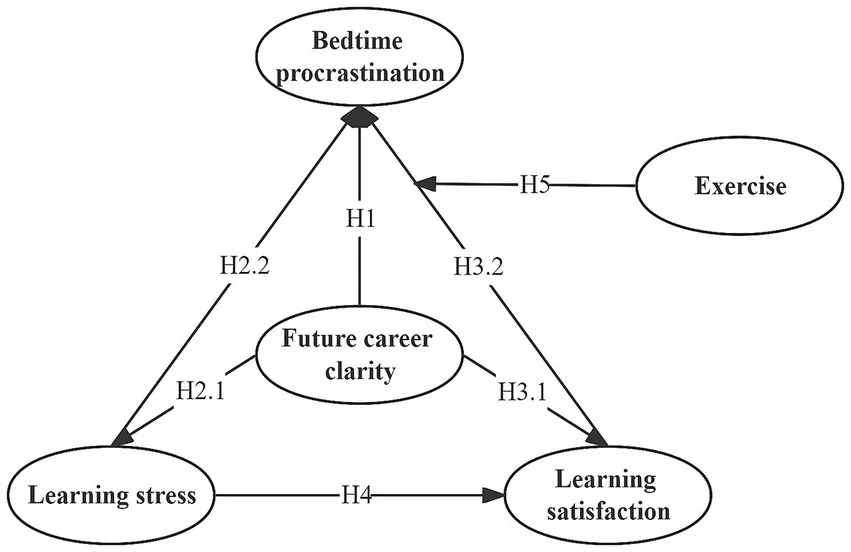
Hypothesized research model. H denotes hypotheses, with the numbers indicating hypothesis numbers.

Based on the aforementioned research limitations and theoretical rationale, this study aims to examine the correlates of bedtime procrastination among college students and proposes the following hypotheses (H1–H5), as depicted in Figure 1:

*H1*: Future career clarity directly and negatively predicts bedtime procrastination.

*H2*: Learning stress mediates the relationship between future career clarity and bedtime procrastination.

*H3*: Learning satisfaction mediates the relationship between future career clarity and bedtime procrastination.

*H4*: Learning stress and learning satisfaction play a chain mediating role in the relationship between future career clarity and bedtime procrastination.

*H5*: Exercise negatively moderates the relationship between learning satisfaction and bedtime procrastination.

Accordingly, grounded in self-regulation theory, this study adopts future career clarity as a measurable proxy for goal setting and proposes a dynamic framework that integrates the three sequential components of monitoring, evaluation, and adjustment. This framework is designed to systematically investigate the correlational structure linking future career clarity, learning stress, learning satisfaction, and bedtime procrastination among university students. At the micro-individual level, this study helps reveal the psychological dynamics underlying college students’ goal setting and behavioral regulation processes, thereby deepening the understanding of factors associated with bedtime procrastination and providing a theoretical basis for sleep hygiene interventions and time management guidance at the individual level. At the macrosocial level, college students’ sleep quality and overall physical and mental health have significant practical implications for enhancing the quality of talent cultivation in higher education and promoting the holistic development of young adults. Taken together, the present study aims to offer theoretical references and practical guidance for alleviating bedtime procrastination and improving sleep health among college students through a multidimensional exploration.

## 2 Method

### 2.1 Research design and data collection

From September to December 2021, we conducted a cross-sectional study among college students in Heilongjiang Province, Northeast China, using an online questionnaire survey combined with respondent-driven sampling. This method involves initially selecting a group of participants meeting the inclusion criteria to serve as “seed” samples. These seed then recommend eligible peers to join the study, forming a first-wave sampling group. When the sampling reaches five to six waves, the sample becomes more representative of the population characteristics. The specific sampling procedure was conducted as follows: first, 13 universities in Heilongjiang Province were selected using purposive sampling; second, 2,000 college students meeting the inclusion criteria were screened from each university; finally, a total of 26,000 college students were identified to participate in the survey. After excluding questionnaires with incomplete information, excessively short completion times, or failed the designated option check, 22,983 valid questionnaires were retained, yielding a response rate of 88.40%. The inclusion criteria were as follows: (1) current enrollment in higher education institutions; (2) provision of informed consent and voluntary participation; and (3) clear self-expression and intact orientation to time, place, and person. Individuals with a reported history of mental illness or cognitive/consciousness disorders were excluded from the study.

### 2.2 Research questionnaire

A structured questionnaire was employed for data collection based on the scales developed by Guan et al. (17). The questionnaire consisted of five sections, with the specific details as follows.

The first section was the future career clarity scale, revised by Guan et al. (17). This scale comprised four items, rated on a 7-point Likert scale (1 = strongly disagree, 7 = strongly agree). The total score was calculated by summing the scores for each item. Higher scores indicated a higher level of self-focused attention on future career development. The Cronbach’s *α* coefficient for this scale in the present study was 0.949.

Second, bedtime procrastination was assessed using the scale developed by Kroese et al. (3). This scale had a single-factor structure, consisting of nine items rated on a 5-point Likert scale (1 = never, 5 = always), with items 2, 3, 7, and 9 being reverse-scored. Item scores were summed to yield a total score, with higher values indicating a stronger tendency toward bedtime procrastination. The Cronbach’s *α* coefficient for this scale was 0.847.

The third section evaluated the students’ learning satisfaction. Given the conceptual clarity of this variable and methodology evidence showing no significant differences in reliability and validity between single-item measures and multi-item scales (18–20), this study used the item, “Overall, are you satisfied with your learning and living conditions?” for measurement. This item was rated on a 5-point Likert scale (1 = extremely dissatisfied, 5 = extremely satisfied), where higher scores reflecting greater learning satisfaction. Previous research supports the reliability and validity of such single-item measures (21).

Next, the fourth section gauged learning stress. Similarly, based on the conceptual clarity of this variable and the adequate reliability and validity of single-item measures (18–20), this study asked participants “To what extent do you feel that your academic and life stress has affected you over the past 2 months?.” This item was rated on a 6-point Likert scale (1 = extremely low stress, 6 = extremely high stress), with higher scores representing greater learning stress.

Finally, he fifth captured exercise levels. Considering the clarity of variable definitions and the reliability and validity of measurement tools (18–20), this study prompted respondents with the question, “How many hours do you exercise per week?” to measure participants’ exercise volume. Responses were recorded on a 4-point scale (1 = 4 h or less, 2 = 4–7 h, 3 = 7–10 h, and 4 = 10 h or more), with higher scores denoting greater exercise volume. Existing research supports the reliability and validity of such single-item questions in physical activity studies (22).

### 2.3 Data analysis

Python 3.10 were used for data analysis. Harman’s single-factor test was employed to examine common method bias in the sample. Descriptive statistics—including percentages, frequencies, means, and standard deviations—characterized the sample and addressed the research questions concerning bedtime procrastination and its influencing factors among college students.

Assuming data normality, partial correlation analysis was conducted to systematically explore the strength and direction of associations among future career clarity, learning stress, learning satisfaction, and exercise with bedtime procrastination, thereby preliminarily identifying factors linked to this behavior.

Based on the established theoretical framework and research hypotheses, this study employed bootstrapping methods to thoroughly analyze the underlying mechanisms of bedtime procrastination.

## 3 Results

### 3.1 Results of common method bias test

Given the high consistency in respondent homogeneity, measurement environment, and item context between the bedtime procrastination and future career clarity scales, there may be an artificial covariance between the independent and dependent variables. To examine the potential presence of common method bias, this study employed Harman’s single-factor test for statistical analysis. The results revealed three factors with eigenvalues greater than 1, collectively accounting for 71.744% of the total variance. The variance explained by the first factor was 37.563%, which was below the threshold of 40%. Based on these findings, it can be concluded that common method bias does not pose a significant issue in this study.

### 3.2 Descriptive analysis of respondent demographic and sociographic information

As shown in Table 1, the sample of 22,983 respondents consisted of 12,217 males (53.16%), and 10,766 females (46.84%). Regionally, the majority of students originated from urban areas (*n* = 13,721, 59.70%). Regarding academic year, first-year university students represented the largest group (*n* = 12,633, 54.97%). With respect to student leadership experience, the largest group consisted of those who had never held a student leadership position (*n* = 13,922, 60.58%).

**Table 1:** Descriptive analysis of demographic sociological information.

Item	Category	Number (*n* = 22,983)	Percentage (%)
Gender	Male	12,217	53.16
	Female	10,766	46.84
Place of origin	Urban	13,721	59.70
	Rural	9,262	40.30
Grade	First year undergraduate	12,633	54.97
	Second year undergraduate	5,789	25.19
	Third year undergraduate	2,651	11.53
	Fourth year undergraduate	794	3.45
	Fifth year undergraduate	30	0.13
	Postgraduate	933	4.06
	Doctorate	153	0.67
Student position	Holds a student position currently	7,850	34.16
	Does not hold a student position currently	13,922	60.58
	Held a student position previously	1,211	5.26
Exercise	4 h or less	12,709	55.30
	4–7 h	6,430	27.98
	7–10 h	2,152	9.36
	10 h or more	1,692	7.36
Learning satisfaction	Extremely unsatisfied	318	1.38
	Unsatisfied	1,196	5.20
	Moderately	8,823	38.39
	Satisfied	9,077	39.49
	Extremely satisfied	3,569	15.54
Learning stress	Extremely low stress	2,177	9.47
	Not excessive stress	4,217	18.35
	Low stress	8,363	36.39
	High stress	6,122	26.64
	Excessive stress	1,497	6.51
	Extremely high stress	607	2.64

In terms of physical exercise, 55.30% of the sample (*n* = 12,709) reported exercising less than 4 h per day. Regarding learning satisfaction, the highest proportion was found among those who reported being satisfied with their learning (*n* = 9,077, 39.49%). In terms of learning stress, the largest group comprised those who rated their academic stress as relatively low (*n* = 8,363, 36.39%).

### 3.3 Partial correlation analysis of different variables

Descriptive statistic for the core variables yielded the following means and standard deviations: bedtime procrastination (26.02 ± 7.58), future career clarity (19.12 ± 5.13), learning satisfaction (3.63 ± 0.86), learning stress (3.10 ± 1.15), and exercise (1.69 ± 0.92).

To exclude the potential influence of the four variables (gender, place of origin, academic year, and student status) on bedtime procrastination, this study conducted partial correlation analyses among future career clarity, learning stress, learning satisfaction, exercise volume, and bedtime procrastination, while controlling for the aforementioned variables.

The results presented in Table 2 indicated that all reported correlations were statistically significant at the *p* < 0.001 level. Specifically, future career clarity was negatively correlated with learning stress (*r* = −0.184) and bedtime procrastination (*r* = −0.282), but positively correlated with learning satisfaction (*r* = 0.393) and exercise (*r* = 0.164). Conversely, learning stress exhibited negative association with learning satisfaction (*r* = −0.398) and exercise (*r* = −0.126), alongside a positive correlation with bedtime procrastination (*r* = 0.263). Furthermore, learning satisfaction was inversely related to bedtime procrastination (*r* = −0.325) and positively linked to exercise (*r* = 0.185). Finally, bedtime procrastination was negatively correlated with exercise (*r* = −0.167).

**Table 2:** Partial correlation analysis among different variables.

Variables	Future career clarity	Learning stress	Learning satisfaction	Bedtime procrastination	Exercise
Future career clarity	–				
Learning stress	−0.184^***^	–			
Learning satisfaction	0.393^***^	−0.398^***^	–		
Bedtime procrastination	−0.282^***^	0.263^***^	−0.325^***^	–	
Exercise	0.164^***^	−0.126^***^	0.185^***^	−0.167^***^	–
Mean	19.12	3.10	3.63	26.02	1.69
Standard deviation	5.128	1.147	0.855	7.579	0.919

***p < 0.001.

### 3.4 Mediating roles of learning satisfaction and learning stress and the moderating role of exercise

#### 3.4.1 Model hypothesis testing

**Figure 2:**
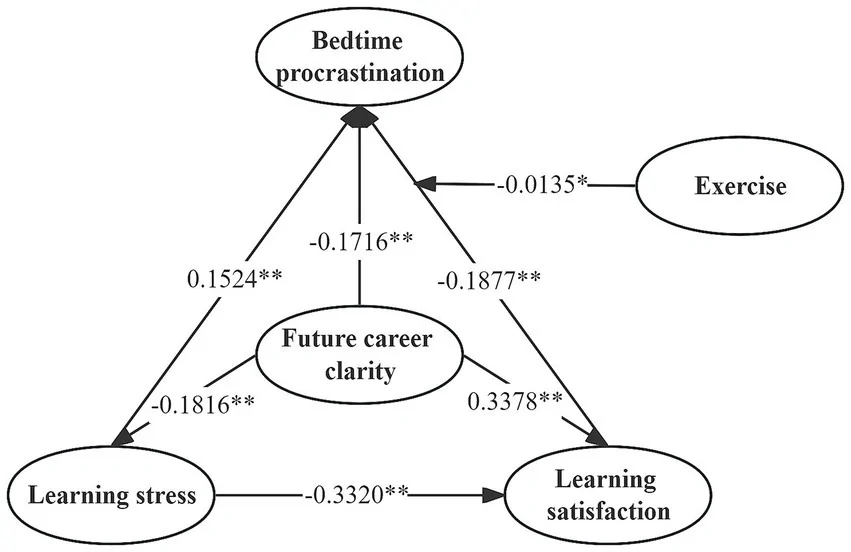
Results of standardized regression weights. **p < 0.01, *p < 0.05.

The results of the model hypothesis testing indicated that all proposed pathways in this study were significant. Therefore, all hypotheses proposed in this study were supported. The specific path coefficients are presented in Table 3, and the model is shown in Figure 2.

**Table 3:** Pathways of influence on bedtime procrastination among college students.

Path relationships	β	*p*	Hypothesis	Result
Future career clarity → Bedtime procrastination	−0.1716	<0.01	H1	Yes
Future career clarity → Learning stress	−0.1816	<0.01	H2.1	Yes
Learning stress → Bedtime procrastination	0.1524	<0.01	H2.2	Yes
Future career clarity → Learning satisfaction	0.3378	<0.01	H3.1	Yes
Learning satisfaction → Bedtime procrastination	−0.1877	<0.01	H3.2	Yes
Learning stress → Learning satisfaction	−0.3320	<0.01	H4	Yes
EXLE → Bedtime procrastination	−0.0135	0.0228	H5	Yes

“EXLE” denotes the interaction term between exercise and learning satisfaction.

#### 3.4.2 Evaluation of the mediating roles of learning stress and learning satisfaction

Bootstrapping was employed to test the sequential mediating roles of learning satisfaction and learning stress between future career clarity and bedtime procrastination. The results are presented in Table 4. First, the results showed that the 95% confidence interval for the total indirect effect did not contain zero (LLCI = −0.1092, ULCI = −0.0958), indicating the presence of a mediating pathway, with a total indirect effect size of −0.1024. Second, after controlling for the mediating variables, the direct effect of future career clarity on bedtime procrastination was tested. The results showed that the 95% confidence interval for the direct effect did not contain zero (LLCI = −0.1863, ULCI = −0.1571), denoting that the direct relationship between future career clarity and bedtime procrastination was statistically significant.

**Table 4:** Mediating path analyses of learning satisfaction and learning stress between future career clarity and bedtime procrastination.

Effect	Path	Confidence interval (95%)	Effect size
Direct effect	Future career clarity → Bedtime procrastination	[−0.1863,−0.1571]	−0.1716
Intermediary effect	Future career clarity → Learning stress → Bedtime procrastination	[−0.0311,−0.0245]	−0.0277
	Future career clarity → Learning satisfaction → Bedtime procrastination	[−0.0688,−0.0580]	−0.0634
	Future career clarity → Learning stress → Learning satisfaction → Bedtime procrastination	[−0.0127,−0.0100]	−0.0113
Total indirect effect	/	[−0.1092,−0.0958]	−0.1024
Total effect	/	[−0.2878,−0.2603]	−0.2740

#### 3.4.3 Effect size assessment

To evaluate the magnitude of the specific predictive paths, we computed Cohen’s *f^2^* effect sizes for each predictor in the nested regression models.

The local effect size of future career clarity on learning stress was *f*^2^ = 0.03, indicating a small effect, whereas its impact on learning satisfaction was *f*^2^ = 0.14, which approached the medium-effect threshold (0.15), reflecting non-negligible practical significance. After controlling for the independent variables, the unique contribution of learning satisfaction to bedtime procrastination yielded *f^2^* = 0.03, suggesting a small effect.

For the supplementary chain-mediated paths, the effect of learning stress on learning satisfaction (controlling for future career clarity) was *f*^2^ = 0.14, which approached the medium-effect threshold (0.15), reflecting non-negligible practical significance. The effect size analysis indicated that learning stress had a small effect on bedtime procrastination (*f*^2^ = 0.02). Furthermore, after controlling for other variables, exercise also demonstrated a small effect on learning satisfaction (*f*^2^ = 0.02). Although most effect sizes were modest, the bootstrap 95% confidence intervals of the indirect effects did not contain zero, corroborating the statistical robustness of the mediation paths.

#### 3.4.4 Evaluation of the moderating role of exercise

**Figure 3:**
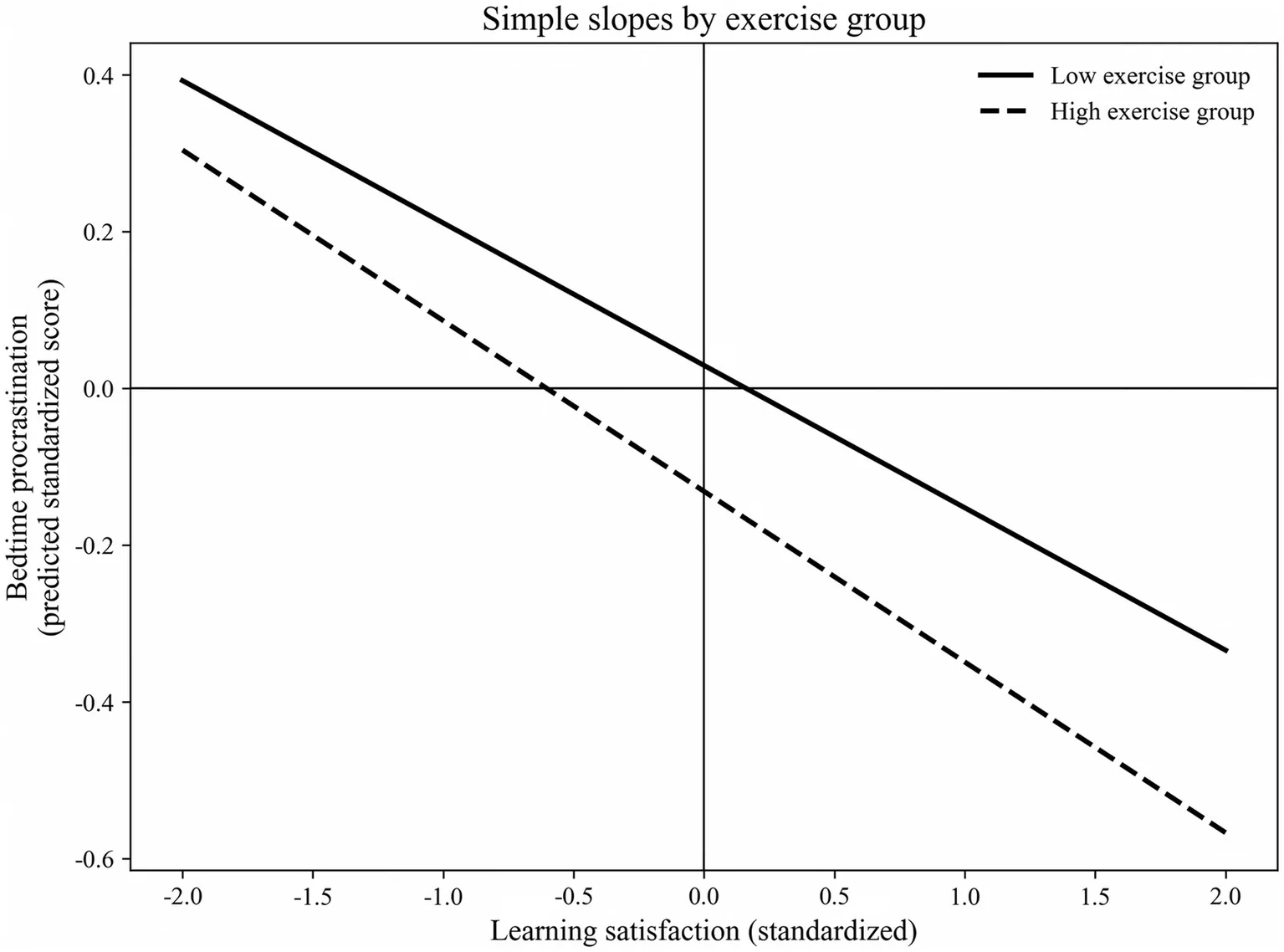
Simple slope test plot.

As shown in Figure 2, exercise significantly moderated the relationship between learning satisfaction and bedtime procrastination (*β* = −0.0135, *p* < 0.05). To further elucidate the pattern of the moderating effect, exercise was divided into high and low groups based on mean ± standard deviation for significance testing, and a simple slopes plot was generated (Figure 3). The results indicated that the moderating effect was statistically significant across different exercise levels. In the low exercise group, learning satisfaction exerted a significant negative predictive effect on bedtime procrastination (simple slopes = −0.1816, LLCI = −0.1966, ULCI = −0.1667), demonstrating that higher learning satisfaction was associated with a lower tendency toward bedtime procrastination. In the high exercise group, learning satisfaction also demonstrated a significant negative predictive effect on bedtime procrastination (simple slopes = −0.2177, LLCI = −0.2467, ULCI = −0.1887), with the absolute value of the coefficient being larger than that in the low exercise group. These findings suggest that the negative effect of learning satisfaction on bedtime procrastination was more pronounced at higher exercise levels; as learning satisfaction increased, the reduction in bedtime procrastination became more evident among individuals with higher exercise levels. The simple slopes plot (Figure 3) visually presents this dynamic pattern. Further analysis of conditional indirect effects revealed that the indirect effect size was −0.1000 (LLCI = −0.1072, ULCI = −0.0929) in the low exercise group and −0.1143 (LLCI = −0.1276, ULCI = −0.1017) in the high exercise group, with the indirect effects being statistically significant across different exercise levels. The results showed that the confidence intervals for the differences among the low-, and high-exercise subgroups did not contain zero, confirming significant differences in the indirect effects across groups with different exercise levels.

## 4 Discussion

Currently, Chinese university students face multiple challenges including high academic pressure and low learning satisfaction. Consequently, addressing bedtime procrastination may benefit not only students’ academic performance and daily functioning but also broader efforts to promote health and educational outcomes among university students. Grounded in self-regulation theory and adopting the perspective of goal-setting imbalance, this study systematically examines the relational patterns among emotional monitoring, evaluation, and bedtime procrastination. It focuses on analyzing the mediating roles of learning stress and learning satisfaction in the relationship between future career clarity and bedtime procrastination, alongside the moderating effect of exercise on the influence of learning satisfaction on bedtime procrastination. In addition, the study innovatively incorporated learning stress and learning satisfaction as core elements of emotional monitoring and evaluation, providing an in-depth analysis of how university students adjust to and cope with negative behaviors triggered by goal-setting imbalance.

### 4.1 Current status of bedtime procrastination among college students

Bedtime procrastination has become an important issue in the field of sleep problems among college students. In this study, the overall score for bedtime procrastination among college students was 26.02 ± 7.58, which was similar to the result of a population-based study in Japan (26.71 ± 7.50). This study also showed that younger participants exhibited a stronger tendency toward procrastination than older participants, which is consistent with the findings of this study (23). Furthermore, multiple literature have indicated that procrastination is essentially a manifestation of self-regulation failure (24). The factors inducing this phenomenon mainly include the following two aspects. First, smartphone use: relevant research has pointed out that the convenience of smartphones can bring users temporary satisfaction and happiness, leading to excessive use that consequently delays sleep onset (25). Second, task delay: postponing academic duties leads to compensatory behaviors before bedtime, accompanied by negative emotions, including sleep resistance, fear, depression, anxiety, and stress (26).

### 4.2 Future career clarity deficit leading to goal setting imbalance

Future career clarity reflects an individual’s self-awareness and visualization of career planning (27). This study focused on the relationship between future career clarity and bedtime procrastination, providing empirical evidence for the field of career planning. The results revealed a significant negative correlation between future career clarity and bedtime procrastination, which is consistent with existing research findings on goal management and procrastination behavior (28). A well-defined career plan helps college students clarify their learning objectives and career expectations, enabling them to possess a stronger sense of purpose and intrinsic drive in their daily academic tasks. This intrinsic motivation can reduce procrastination behavior caused by a lack of clear goals, thereby alleviating bedtime anxiety and preventing sleep delay (29).

Therefore, career planning helps college students establish personal goals and clarifies their current and future developmental directions. Such career guidance can assist them in resolving potential cognitive biases related to career choices and academic planning, allowing them to identify their strengths and enhance their competitiveness (30). For universities, career guidance contributes to promoting teaching reform, improving student learning satisfaction, and enhancing their institutional reputation. Addressing career planning issues is not merely an individual or family concern; it profoundly affects the social reputation of universities and even holds significant implications for national political and economic development, as well as social harmony and stability.

### 4.3 Emotional monitoring and evaluation of learning stress and learning satisfaction

The results indicate that future career clarity significantly and negatively predicts bedtime procrastination, with learning stress and satisfaction playing mediating roles. This suggests that college students with a clearer future career direction exhibit lower levels of bedtime procrastination, and this effect may be jointly realized through reduced learning stress and enhanced learning satisfaction.

From the perspective of self-regulation theory, individuals continuously adjust their behavior around goal setting, process monitoring, and outcome evaluation. When college students have a high level of clarity about their future career development, their learning goals and developmental directions become more specific, making it easier for them to connect their current learning to future development. This connection reduces stress associated with goal ambiguity and enhances positive evaluations of the learning process. Conversely, when individuals lack a clear direction in future career planning, they are more prone to goal-setting imbalance, accompanied by higher learning stress and lower learning satisfaction, which in turn increases the likelihood of bedtime procrastination.

This finding is consistent with those of similar studies. Existing research has shown that career development-related resources, such as career adaptability and competence, are closely associated with students’ learning engagement (31), life satisfaction, and academic performance (32). Career adaptability can also influence college students’ life satisfaction through academic engagement, suggesting that a clear future development orientation helps improve individuals’ current psychological learning experiences. Academic stress significantly affects student satisfaction, with higher academic stress generally accompanied by lower satisfaction (33). Therefore, the findings of this study regarding the influence of future career clarity on bedtime procrastination through learning stress and satisfaction are supported by existing literature.

Notably, the indirect effect size for the link between academic stress and learning satisfaction in this study was relatively small, which reflects the complexity of the relationship between these two constructs. This can be explained from several perspectives, as follows. First, drawing on self-regulation theory, the formation of learning satisfaction does not depend solely on the reduction of stress levels; rather, it derives more from an individual’ s comprehensive cognitive appraisal of goal setting regarding future career planning. Although the alleviation of academic stress may create a favorable emotional context for enhancing satisfaction, a simple decrease in stress does not necessarily translate directly into higher satisfaction, and its independent explanatory power for satisfaction is inherently limited. Second, the existing empirical evidence indicates that the mediating effects between academic stress and learning satisfaction typically range from −0.10 to −0.20, falling within the small-effect category, which objectively constrains the overall magnitude of the serial indirect effect that involves this specific link (34). Moreover, the cross-sectional design may have underestimated the dynamic cumulative effects of stress and satisfaction over time, thereby limiting the estimation of effect sizes. In summary, although the indirect effect of academic stress on the association with learning satisfaction is modest, its directional pattern within the serial mediation model is statistically consistent with theoretical expectations regarding the relationship between future career clarity and bedtime procrastination.

Overall, this study demonstrates that a well-defined career vision not only directly affects bedtime procrastination among college students but also influences their bedtime behaviors through the combined effects of learning stress and learning satisfaction. This provides a reference for universities to implement interventions in areas such as career education, stress management, and learning.

### 4.4 The moderating effect of active exercise on behavioral empowerment

The results indicate that exercise significantly moderates the relationship between learning satisfaction and bedtime procrastination. In other words, by regulating an individual’s exercise level, the extent to which learning satisfaction influences bedtime procrastination can be altered effectively. This finding is consistent with the existing research. Previous studies have indicated that sustained exercise is closely associated with improved mental health (35). Moreover, exercise not only directly promotes physical and mental health, but also optimizes learning satisfaction by enhancing individual self-efficacy, thereby achieving behavioral empowerment and reducing bedtime procrastination (36). Therefore, when individuals experience a goal-setting imbalance in future career planning, the accompanying negative monitoring and evaluation effects such as high learning stress and low learning satisfaction further influence bedtime procrastination. The high self-efficacy brought about by moderate exercise enables individuals to adopt proactive, goal-oriented behavioral strategies when facing learning challenges, rather than resorting to avoidance or procrastination.

As a moderating variable, exercise significantly moderated the relationship between learning satisfaction and bedtime procrastination in both the low exercise group and the high exercise group, and the negative effect of learning satisfaction on bedtime procrastination was more pronounced in the high exercise group. Compared to low exercise levels, higher exercise volume was associated with a stronger negative predictive effect of learning satisfaction on bedtime procrastination. In the low exercise group, due to the lack of regular physical exercise, individuals were more likely to engage in bedtime procrastination when facing similar learning situations, and learning satisfaction was significantly negatively correlated with bedtime procrastination. In the high-exercise group, individuals benefited from the multiple advantages of exercise, which effectively weakened the negative impact of learning satisfaction on sleep. Under the same level of stress, individuals in the high exercise group were less likely to exhibit bedtime procrastination or their degree of procrastination was milder. As a highly targeted and relatively cost-effective intervention, exercise has provided empirical evidence and practical guidance for reducing bedtime procrastination among students. However, this study also suggests that higher levels of daytime fatigue may influence bedtime procrastination (30, 37).

Based on these findings, educational and health intervention strategies should incorporate moderate exercise into policymaking and resource allocation, creating suitable exercise environments and opportunities for students, emphasize physical activity, and at the same time, moderate rather than excessive exercise. Currently, the primary methods used by universities to promote student exercise include mandatory running check-ins and physical fitness tests. In the future, diverse forms and types of physical activity should be developed, such as sports clubs and onsite gymnastics training, so that students can experience the joy of exercise through active participation, thereby making it easier for them to develop consistent exercise habits and ultimately enhance their academic success, career development, and overall health.

### 4.5 Limitations and future directions

Although this study provides a preliminary exploration of the relationship between future career clarity and bedtime procrastination among college students in Northeast China, the regional specificity of the sample limits the generalizability of the findings. Future research should extend to other regions or international contexts to enhance the external validity of the conclusions. Second, this study used single-item measures to assess learning stress and satisfaction, which may not have fully captured the complexity of the two psychological constructs. Drawing on prior methodological frameworks (38, 39), future studies should consider employing multidimensional scales to assess the different dimensions of learning stress and satisfaction, thereby enabling a more detailed and comprehensive understanding and analysis of these two core variables. Furthermore, the cross-sectional design of this study precludes strict causal inferences among variables. Future research should adopt a longitudinal follow-up or experimental design to further clarify the causal directions among variables.

## 5 Conclusion

Bedtime procrastination is prevalent among Chinese college students. However, research on the relationships between future career clarity, learning satisfaction, learning stress, and bedtime procrastination in this population remains insufficient. This study empirically demonstrates that a deficit in future career planning disrupts goal-setting balance, which exacerbates learning stress, diminishes learning satisfaction, and ultimately triggers bedtime procrastination. Crucially, moderate physical exercise acts as a protective buffer; it boosts self-efficacy and empowers students to adopt proactive coping strategies rather than resorting to sleep delay. Based on these findings, there is an urgent need to construct an integrated intervention framework. At the macro level, this study responds to the Healthy China Strategy’s concern for the physical and mental health of young people, providing a theoretical basis for universities to promote the deep integration of career planning education and health promotion. At the micro level, this study reveals the transmission pathway among goal setting, emotional monitoring, and behavioral adjustment, offering empirical support for the implementation of precise interventions.

## Data Availability

The raw data supporting the conclusions of this article will be made available by the authors, without undue reservation.
